# Bulk RNA sequencing analysis of developing human induced pluripotent cell-derived retinal organoids

**DOI:** 10.1038/s41597-022-01853-x

**Published:** 2022-12-09

**Authors:** Devansh Agarwal, Rian Kuhns, Christos N. Dimitriou, Emmalyn Barlow, Karl J. Wahlin, Ray A. Enke

**Affiliations:** 1 Viterbi Family Department of Ophthalmology at the Shiley Eye Institute, University of California San Diego, La Jolla, CA 92093 USA; 2 Department of Bioengineering, University of California San Diego, La Jolla, CA 92093 USA; 3 Department of Biology, James Madison University, Harrisonburg, VA 22807 USA; 4 The Center for Genome & Metagenome Studies, James Madison University Harrisonburg, Harrisonburg, VA 22807 USA

**Keywords:** Molecular neuroscience, Gene regulatory networks

## Abstract

Retinogenesis involves the transformation of the anterior developing brain into organized retinal lamellae coordinated by intricate gene signalling networks. This complex process has been investigated in several model organisms such as birds, fish, mammals and amphibians, yet many facets of retinal development are different in humans and remain unexplored. In this regard, human pluripotent stem cell (hPSC)-derived 3D retinal organoids and Next Generation Sequencing (NGS) have emerged as key technologies that have facilitated the discovery of previously unknown details about cell fate specification and gene regulation in the retina. Here we utilized hPSCs integrated with fluorescent reporter genes (SIX6-p2A-eGFP/CRX-p2A-h2b-mRuby3) to generate retinal organoids and carry out bulk RNA sequencing of samples encompassing the majority of retinogenesis (D0-D280). This data set will serve as a valuable reference for the vision research community to characterize differentially expressed genes in the developing human eye.

## Abstract (summary)

**Table:** 

Measurement(s)	mRNA Sequencing
Technology Type(s)	Next Generation Sequencing
Factor Type(s)	Tissue Source • Tissue Type
Sample Characteristic - Organism	Homo sapiens
Sample Characteristic - Environment	cell culture

## Background & Summary

Retinal development entails the complex interplay between spatiotemporal gene expression and regulatory events, yet many of their mechanistic interactions are not understood properly. Even though retinogenesis has been investigated from a transcriptomic perspective in several model organisms such as fish, amphibians, birds, and mammals, the extent to which it has been studied in humans is quite limited^1–4^. Comparative studies utilizing human pluripotent stem cell (hPSC)-derived mini-retinas have implied that *in vitro* gene expression parallels *in vivo* development of the eye^5^. These investigations have been made possible by the emergence and commercialization of Next Generation Sequencing (NGS) technologies such as RNA-seq that have facilitated the characterization of global transcriptomic differences across multiple tissue types in addition to cell-specific transcript isoforms^6^.

Recent cutting-edge breakthroughs in the reprogramming of human somatic cells into induced PSCs and their subsequent differentiation into 3D retinal tissues have greatly facilitated the investigation of retinal development in real-time as well as the study of various retinopathies^7,8^. Several landmark publications have demonstrated that 3D organoids derived from hPSCs have a laminar-organized retina with each major class of retinal cells, including rod and cone photoreceptors^9–11^. To generate retinal organoids, stem cells are aggregated in conditions that favor their differentiation into neuroectodermal lineages via two main methods: forced aggregation or embryoid body formation. As the 3D tissue matures, its structures spontaneously start expressing eyefield expressed transcription factors (e.g., SIX6). Later, these eye field structures develop into VSX2 positive optic vesicles as well as MITF expressing retinal pigmented epithelium (RPE)^12–14^. Following manual excision of optic vesicles, organoids can be maintained for extended periods during which time they develop into laminar-organized retinal structures similar to those found *in vivo*. Similar to retinogenesis *in vivo*, retinal organoids show a differentiation wave that follows the posterior to anterior (central to peripheral) wave that not only mimics *in vivo* development but also recapitulates major cellular and molecular hallmarks^15–18^. Thus, the generation of retinal organoids presents the unique opportunity of testing hypotheses that were limited to animal models or 2D cell cultures and can help complement approaches to drug screening. Simultaneously, they can provide novel insights into human biology and bridge the gap between animal studies and clinical trials^7,19^.

In the current study we carried out detailed temporal analyses of developing human retinas generated from hPSCs integrated with fluorescent reporter genes indicative of the eyefield (SIX6-eGFP) and photoreceptor lineage (CRX-h2b-mRuby3) by using high quality total RNAs extracted from human retinal organoids at varying stages of development from early optic development to photoreceptor maturation. While other protocols exist that may result in some transcriptional differences, our use of retinal reporters to create a dataset spanning the majority of retinal development and maturation (D0-D280) will serve as a reliable reference for future studies of the human eye.

## Methods

### Stem cell line maintenance and processing

A human induced pluripotent stem cell (hiPSC)-derived 3D retinal organoid system that mimics fetal human retinal development was used as previously described with authorization from the UC San Diego Institutional Review Board Committee^9,12,20^. The IMR90.4 iPSC line was obtained from WiCell (Madison, WI). Cells were routinely tested for mycoplasma by PCR^21^. Pluripotency of cells was evaluated with antibodies for NANOG, OCT4, SOX2, SSEA4. Stem cells were maintained antibiotic free on 1% (vol/vol) Matrigel-GFR (#354230; Corning, New York, NY) coated dishes at 37 °C under hypoxic conditions (10% CO_2_/5% O_2_) in mTeSR1 (Stem Cell Technologies, Vancouver, Canada)^9,22–24^. Cells were passaged every 4–6 days, with Accutase (#A6964; Sigma, St. Louis, MO) for 8–10 minutes, dissociated to single cells, quenched with mTeSR1 plus 5 μM (−) blebbistatin (B; #B0560; Sigma, St. Louis, MO), pelleted at 80 × g for 5 minutes, resuspended in mTeSR1 + B and plated at 5,000 cells per 35 mm dish^25^. After 48 hours, cells were fed without B.

### Human retinal organoid differentiation

**Fig. 1:**
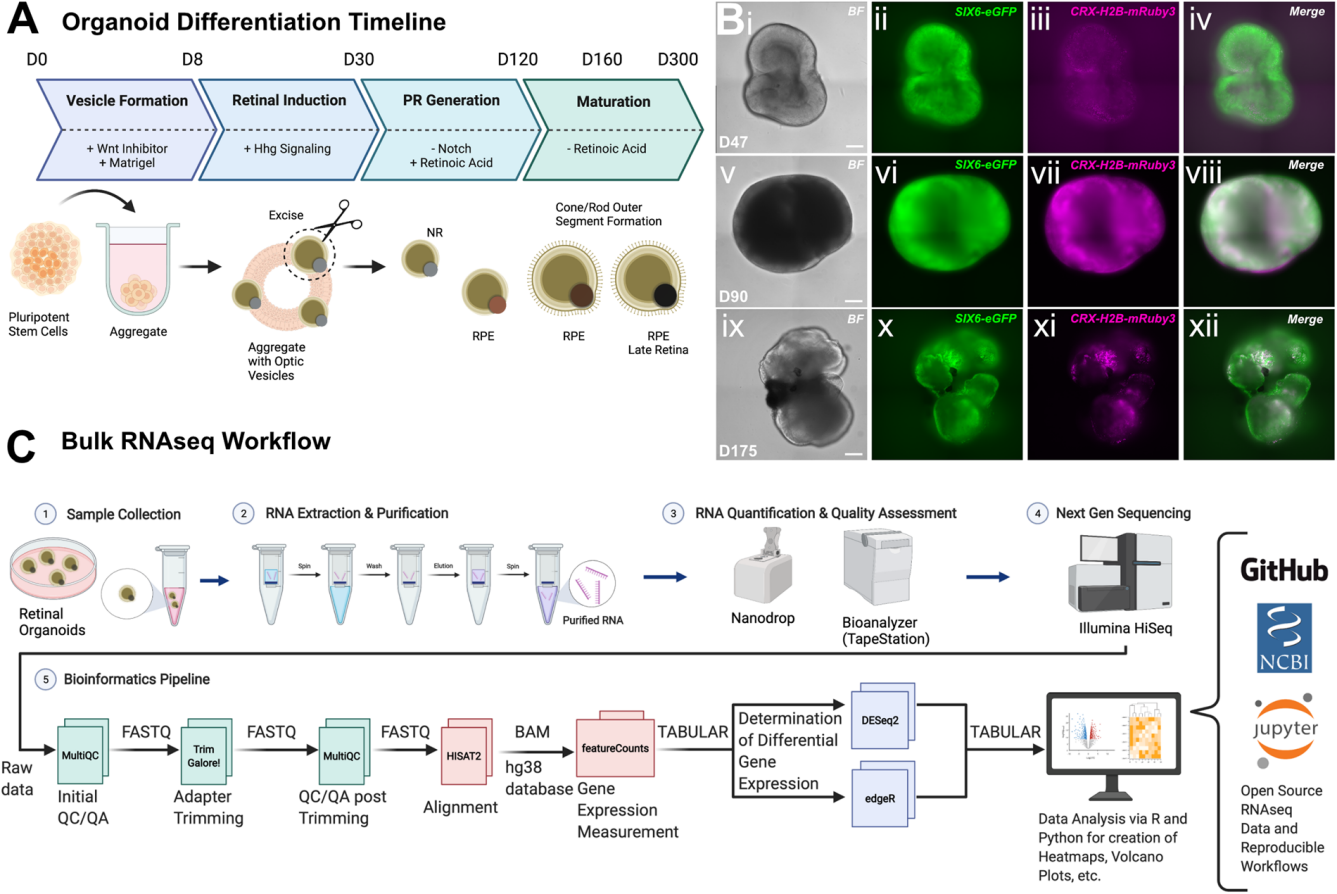
Overview of the hiPSC-derived culture system and experimental workflow. (**A**) Schematic diagram of the timeline for organoid differentiation from forced aggregation of hPSCs until the formation of 3D retina cups with photoreceptor outer segments. (**B**) Live fluorescent micrographs of representative retinal organoids at D47 (i-iv), D90 (v-viii) and D175 (ix-xii) showcasing brightfield (BF), SIX6-eGFP (green), CRX-h2b-mRuby3 (magenta) and Merge at each time point respectively. Scale bar for D47/90/175 is 150 µm. (C) Flowchart highlighting the bulk RNA sequencing workflow including sample collection, RNA extraction/purification, quantification/quality assessment, next generation sequencing and data analysis using open source bioinformatics tools.

hiPSCs and hiPSC-derived retinal organoids were cultured as previously described (Fig. 1a)^9^. Multiple batches of cell lines were grown in tandem for experimental replication. To ensure that organoids collected for sequencing were bonafide retinas we used a SIX6-p2A-eGFP (SIX6-eGFP)/CRX-p2A-h2b-mRuby3(CRX-h2b-mRuby3) dual color reporter line similar to that described in our earlier publication (Fig. 1b)^12,26^. Briefly, on day 0 (D0) stem cells were passaged with Accutase for 12 min and 1,000 cells in 50 μl’s of mTeSR1 + B were seeded per well into a polystyrene 96-well U-bottom plate (#650180; Greiner, Frickenhausen, Germany). Over the first 4 days, aggregates were transitioned to neural induction medium (BE6.2-NIM) by adding 50 μl BE6.2 + 2% MG on day 1 (D1) and 50 μl BE6.2 + 1% MG each day thereafter. On days 4–8 (D4–8) a 50% medium exchange (100 μL) was performed daily and every other day thereafter. NIM also contained 3 μM of the WNT antagonist (IWR-1-endo; #681669 EMD Millipore, Burlington, MA) from D1-6. For hypoxia experiments, feeding occurred in ambient air for approximately 5 minutes and returned to hypoxia for growth. Organoids were grown in BE6.2 + 300 nM Smoothened agonist (SAG; #566660; EMD Millipore, Burlington, MA) from D8-D14 and then LTR + SAG from D14-D18. For longer term experiments we used sharpened tungsten needles to excise optic vesicles from D10-12 as previously described^9^. Organoids were maintained in suspension in LTR medium at low density (<24–36/10 cm untreated polystyrene petri dish) and fed every 2–3 days. Poorly defined vesicles were periodically removed. To increase survival and differentiation, 500 nM all-trans retinoic acid (ATRA; #R2625; Sigma, St. Louis, MO) was added to LTR from D20 and 10 μM DAPT (#565770; Calbiochem, San Diego, CA) from D28–42.

### Total RNA isolation

Total RNA was extracted from 28 hiPSCs and hiPSC-derived retinal organoids using a Qiagen AllPrep Mini Kit (Hilden, Germany) with an on-column DNaseI treatment step per the manufacturer’s instructions (Table 1). Isolated RNAs were eluted in nuclease free water, validated for quality and quantity using UV spectrophotometry, and stored at −80 °C. RNAs with an OD260/280 ratio between 1.9 and 2.1 were deemed high quality and used for downstream analysis (Fig. 1c).

**Table 1:** RNA-seq samples, read/alignment metrics, and public SRA accessions.

Time Point	Sample #	Read Length (bp)	Million Read-pairs	% Alignment	NCBI SRA Data Accession #
Day 0	1	2 × 150	31.1	83.8	SRR15435654
Day 0	2	2 × 150	26.1	86.7	SRR15435653
Day 0	3	2 × 150	25.6	81.8	SRR15435642
Day 10	3	2 × 150	30.7	84.9	SRR15435633
Day 10	4	2 × 150	27.3	83.9	SRR15435632
Day 10	5	2 × 150	28.1	83.8	SRR15435631
Day 10	6	2 × 150	28.3	84.9	SRR15435630
Day 25	1	2 × 150	30.7	81.4	SRR15435645
Day 25	2	2 × 150	30.7	82.7	SRR15435644
Day 25	3	2 × 150	27.1	83.7	SRR15435643
Day 25	4	2 × 150	28.5	85.0	SRR15435641
Day 65	2	2 × 150	27.1	85.1	SRR15435637
Day 65	3	2 × 150	28.0	79.8	SRR15435636
Day 65	5	2 × 150	26.0	80.9	SRR15435635
Day 65	6	2 × 150	28.6	82.4	SRR15435634
Day 100	3	2 × 150	28.1	82.2	SRR15435629
Day 100	4	2 × 150	25.6	83.9	SRR15435628
Day 100	5	2 × 150	26.1	84.5	SRR15435627
Day 100	6	2 × 150	26.4	85.7	SRR15435652
Day 180	1	2 × 150	28.4	86.0	SRR15435651
Day 180	2	2 × 150	25.9	81.3	SRR15435650
Day 180	3	2 × 150	27.3	83.8	SRR15435649
Day 180	4	2 × 150	28.2	81.3	SRR15435648
Day 180	5	2 × 150	28.1	81.4	SRR15435647
Day 180	6	2 × 150	26.7	78.1	SRR15435646
Day 280	A2	2 × 150	28.5	83.9	SRR15425640
Day 280	B1	2 × 150	27.6	86.2	SRR15425639
Day 280	C1	2 × 150	30.4	86.9	SRR15425638

### RNA QC, library preparation and HiSeq sequencing

Sample QC, RNA library preparations and sequencing reactions were conducted at GENEWIZ, LLC. (South Plainfield, NJ). The concentration of RNA was quantified using a Qubit Fluorometer (Life Technologies, Carlsbad, CA) and RNA integrity was assayed using a TapeStation (Agilent Technologies, Palo Alto, CA). Samples passing initial QC were prepared for sequencing using a SMART-Seq v4 Ultra Low Input Kit for full-length cDNA synthesis and amplification (Clontech, Mountain View, CA), and an Illumina Nextera XT library (Illumina, San Diego, CA) was used for sequencing library preparations. Briefly, cDNA was fragmented, and an adaptor was added using transposase, followed by limited-cycle PCR to enrich and add index to the cDNA fragments. The final library was assessed with an Agilent TapeStation. Sequencing libraries for the 28 cDNA samples were multiplexed and sequenced using the Illumina HiSeq sequencing platform. 56 PE FASTQ files received back from Genewiz were analyzed using a customized bioinformatics workflow (Fig. 1c).

### Quality validation and read alignment

**Fig. 2:**
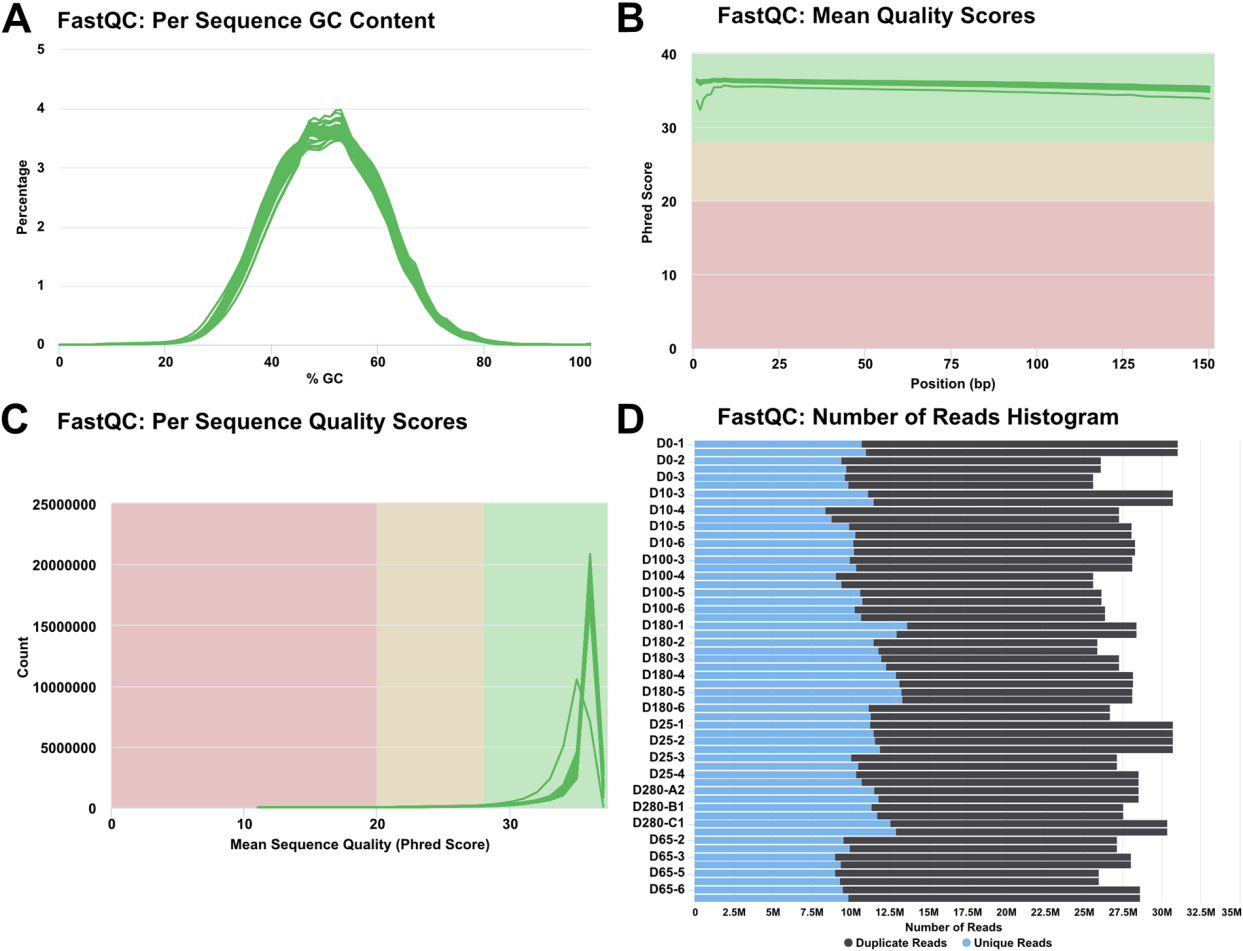
Overview of sequence quality utilizing FastQC and MultiQC. (**A**) Per sequence GC content plot indicating roughly normal GC content of all 28 paired end reads. (**B**) Per base Phred quality scores averaged across each base in the read denoting >33 value in all samples. (**C**) Per sequence Phred quality scores showing that the majority of subsets of the reads have quality >33. (**D**) Raw sequence counts for each sample highlighting duplicate and unique reads.

Between 25.6–31.1 million PE sequence reads per sample were delivered from Genewiz (Table 1). Quality of sequence reads in the 56 FASTQ files was evaluated using FastQC and MultiQC analysis (see Code Availability 1–2), including per sequence GC content (Fig. 2a), per base (Fig. 2b) and per sequence (Fig. 2c) quality analysis, and duplicate read analysis (Fig. 2d) for all reads in the data set^27^. Collectively, Fig. 2 demonstrates that all 56 FASTQ sequencing files were of high quality and suitable for downstream bioinformatics analysis. Sequence reads were aligned to the human hg38 reference transcriptome using the HISAT2 splice-aware aligner (see Code Availability 3)^28^. The percentage of aligned reads ranged from 78.1 to 86.9% (Table 1). Aggregate data visualizations for FastQC and HISAT2 were generated using MultiQC software (see Code Availability 2)^29^.

### Data transformation and downstream analysis

**Fig. 3:**
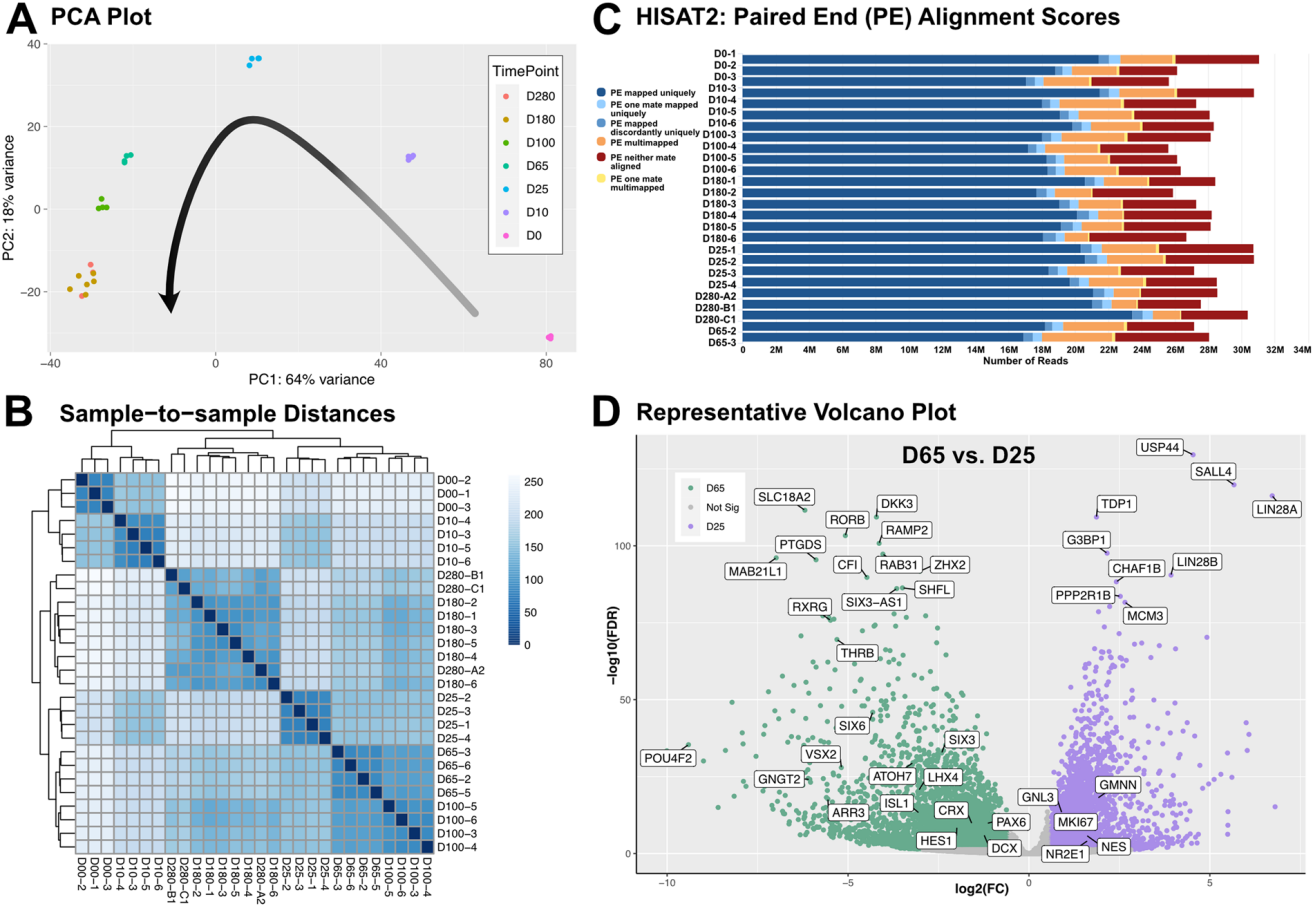
Overview of sampling and sequence alignment quality. (**A**) Principal Component Analysis (PCA) plot showing replicates from time points clustered according to their variance in two dimensions. (**B**) Sample to sample distance heatmap indicating the relative distance between replicates of the same time point and those from different days. (**C**) HISAT2 paired end (PE) alignment histogram denoting majority of the reads with unique mapping in the human reference genome. (**D**) Volcano plot representing significantly differentially expressed genes upregulated at D65 versus D25 graphed with respect to -log10(False Discovery Rate) in the y-axis and log2(Fold Change) in the x-axis.

Transcript alignment of each sample was achieved using HISAT2 with the reference hg38 human genome^28^. HISAT2 outputs were fed into featureCounts for transcript quantification (see Code Availability 4)^30^. Subsequently, the count tables or matrices were input into the DESeq2 statistical package for determination of differential transcript expression between samples (see Code Availability 5)^31^. DESeq2 is available as an R package and was used to generate a principal component analysis (PCA) plot demonstrating the variance between distinct sample groups as well as similarity within sample replicates for all 28 samples (Fig. 3a) as well as the sample to sample distance heatmap showcasing large sample distances between various time points and tight clustering within the same replicates of a particular time point (Fig. 3b). To verify appropriate alignment of transcripts with respect to the reference genome, the alignment files from HISAT2 were added to MultiQC and paired end alignment scores were generated for each sample (Fig. 3c). To specifically highlight the utility of this dataset for studying retinal development, the differential gene expression output table from each time point pair was used for the creation of volcano plots to represent significant gene expression in terms of -log10(False Discovery Rate) versus log2(Fold Change) (Fig. 3d). This analysis demonstrates differential expression of early retinal progenitor transcripts including *LIN28A, NES, MKI67, GMNN and GNL3* at the day 25 time point and maturing retina genes *POU4F2, VSX2, CRX, ATOH7, ARR3 and LHX4* at day 65. These findings are similar to those observed by Hoshino *et al*. in the transcriptomic analysis of the developing human retina derived from fetal tissue supporting the practicality of our data set in studying retinogenisis across a broad range of time points^1^. Collectively, Fig. 3 demonstrates that the sampling strategy used in our study was effective for comparing differential transcript expression in early and maturing retinal organoids.

## Data Records

Raw FASTQ files for the RNAseq libraries were deposited to the NCBI Sequence Read Archive (SRA), and have been assigned BioProject accession # PRJNA754196 (Table 1)^32^. Additionally, processed HISAT2 alignment.bam output data files are accessible from the same BioProject for each of the 28 paired end samples analyzed in our study. The outputs for featureCounts and DESeq2 (.tabular), and R scripts for creation of volcano plots are available publicly at GitHub (see Code Availability).

## Technical Validation

### Quality control-RNA integrity

Quality of total RNA fractions was assessed using an Agilent Tapestation to calculate an RNA Integrity Number (RIN). The RIN algorithm determines the RNA quality of the samples with the highest quality having a score of 10. Conventional to NGS analysis, only RNA samples with a RIN >8 were used for sequencing analysis.

### Quality assessment of sampling, raw sequences, and sequence alignment

FastQC and MultiQC analyses were used to demonstrate that the number and overall quality of raw RNA-seq reads were within the acceptable range for downstream analysis (Fig. 2a–d). Principal component analysis (PCA) and sample-sample distance mapping confirmed the similarity between biological replicates and variability between sample groups (Fig. 3a,b). MultiQC analysis of HISAT2 alignment outputs was used to demonstrate that between 78.1–86.9% of RNA-seq reads were successfully mapped to the human hg38 transcriptome assembly (Fig. 3c and Table 1).

## Usage Notes

The bioinformatics pipeline applied to our data set outlined in Fig. 1c was achieved using a collection of freely available, open access tools. These analyses however, are interchangeable with many other currently available tools for achieving different experimental outcomes. Our raw FASTQ data can be aligned to any available human reference genome or transcriptome, including the most recent GRCh38/hg38 reference assembly using a variety of freely available aligners. In this study we used the splice-aware alignment tool HISAT2, which can be used for traditional differential gene expression analysis or paired with the StringTie and Ballgown softwares for novel isoform analysis^33^. Alternatively, ultrafast and data light alignment-free transcriptome pseudoaligners such as Kallisto, Sailfish and Salmon can be applied to these data with the specific intent of expression quantification of previously characterized mRNA isoforms^34–36^. Alignment-free pipelines significantly reduce the time and computing power required for analysis, but are not suitable for novel isoform analysis. Here our gene quantification and differential gene expression analysis was carried out using the featureCounts and DESeq2 software suites, however other publicly available packages such as StringTie may also be used for similar analysis^33^. Importantly, QC data presented in Figs. 2,3 demonstrates the high quality of sequencing reads and precision of sampling respectively making this data set compatible with alignment tools currently available as well as new alignment tools that may become available in the future.

Our data set will be useful for a variety of studies investigating the developing human retina. In particular, this work will build on existing genomic data sets investigating the developing human retina especially via hPSC-derived 3D retinal organoids. Kim *et al*. generated cone-rich retinal organoids and carried out transcriptomic profiling to validate temporal expression of retinal marker genes and implicate transcripts involved in retinal degeneration^37^. Furthermore, the data can be applied to compare gene regulation across species. For instance, we compared mRNA transcripts derived from embryonic chicken retinas to the RNA-seq reads from the current study and highlighted the conserved nature of transcriptional regulation of CTBP2/RIBEYE^20^. A recent report by Wahlin *et al*. employed a SIX6-GFP/POU4F2-tdTomato dual reporter hPSC line to identify the transcriptomic differences among the human retina, hypothalamus, and midbrain/hindbrain organoids^12^. Notably, Hoshino and colleagues collected their RNA-seq data from whole fetal retinas^1^. On the other hand, the study presented here utilizes hPSCs integrated with eye field and retinal lineage fluorescent reporters to generate 3D organoids thereby providing a comprehensive dataset to the vision research community for studying key molecular and transcriptomic occurrences during retinogenesis.

Several considerations must be taken into account when using these data for downstream analysis. First, RNAs were extracted from hPSCs and hPSC-derived retinal organoids without any further enrichment for cell type. Therefore, the resulting downstream analysis will be representative of heterogeneous mixtures of differing cell types within these organoid tissues. In contrast to our bulk RNA-seq study, several recent studies have applied single cell RNA-seq analysis to similar hPSC-derived 3D retinal organoids. These studies provide differential gene expression analysis at single cell resolution in developing retinal organoids compared to that of developing fetal retinal tissue^38–40^. Second, cDNA libraries were prepared using a poly dT primer, thus the data set is representative of only polyadenylated transcripts and does not represent many non-coding RNA or other non-polyadenylated cellular transcripts. Additionally, usage of poly dT priming introduces a bias towards overrepresentation of the 3′ end of transcripts, particularly in the case of relatively large transcripts. Due to low input amounts of RNA for several of our mature organoid samples, a SMART-seq library preparation was applied. This methodology allowed for retention of all samples of interest in the study with the trade-off of having lower complexity sequencing libraries. Finally, although the quantity of sequenced and mapped reads per sample in this study (Table 1 and Fig. 3a) is sufficient for robust differential transcript/gene expression analysis; it is below the conventional threshold for thorough differential isoform analysis^41^. Taking these considerations into account, these data will be a useful resource for the ophthalmic research field for rigorous and accurate analysis of polyadenylated transcriptional networks in the developing human retina.

## Data Availability

The following open access software and versions were used for quality control and data analysis as described in the main text: 1. FastQC, version 0.11.5 was used for quality analysis of raw FASTQ sequencing data: http://www.bioinformatics.babraham.ac.uk/projects/fastqc/ 2. MultiQC, version 1.11 was used to aggregate and visualize FastQC and HISAT2 data outputs: https://multiqc.info/ 3. HISAT2-index-align, version 2.2.1 was used to index and align sequencing reads to the human hg38 genome: http://daehwankimlab.github.io/hisat2/ 4. featureCounts, version 2.0.3 was used to assign sequence reads to features in the human genome: http://subread.sourceforge.net/ 5. DESeq2, version 1.36 was used to quantify differentially expressed transcripts across various time points and replicates: http://www.bioconductor.org/packages/release/bioc/html/DESeq2.html All code and scripts used for quality assessment and data analysis in this study is available at: https://github.com/WahlinLab/Organoid_RNAseq_SciData22.
